# A comprehensive analysis of the naturally occurring polymorphisms in HIV-1 Vpr: Potential impact on CTL epitopes

**DOI:** 10.1186/1743-422X-5-99

**Published:** 2008-08-23

**Authors:** Alagarsamy Srinivasan, Velpandi Ayyavoo, Sundarasamy Mahalingam, Aarthi Kannan, Anne Boyd, Debduti Datta, Vaniambadi S Kalyanaraman, Anthony Cristillo, Ronald G Collman, Nelly Morellet, Bassel E Sawaya, Ramachandran Murali

**Affiliations:** 1 Thomas Jefferson University, Department of Microbiology and Immunology, Jefferson Alumni Hall Rm 461, 1020 Locust Street, Philadelphia, PA 19107, USA; 2 University of Pittsburgh, Department of Infectious Diseases & Microbiology, Parran Hall Rm 439, 130 DeSoto Street, Pittsburgh, PA 15261, USA; 3 Department of Biotechnology, Indian Institute of Technology Madras, Chennai 600036, India; 4 Wellesley College, 21 Wellesley College Rd Unit 7430, Wellesley, MA 02481, USA; 5 Advanced Bioscience Laboratories, Inc., 5510 Nicholson Lane, Kensington, MD 20895, USA; 6 University of Pennsylvania School of Medicine, 522 Johnson Pavilion, 36th and Hamilton Walk, Philadelphia PA 19104, USA; 7 Unite de Pharmacologie Chimique et Genetique, INSERM, Avenue de l'Observatoire, Paris Cedex 06, France; 8 Department of Neuroscience, Center for Neurovirology, Temple University School of Medicine, Philadelphia, PA 19122, USA; 9 University of Pennsylvania School of Medicine, Dept of Pathology and Laboratory Medicine, 243 John Morgan, Philadelphia PA 19104, USA

## Abstract

The enormous genetic variability reported in HIV-1 has posed problems in the treatment of infected individuals. This is evident in the form of HIV-1 resistant to antiviral agents, neutralizing antibodies and cytotoxic T lymphocytes (CTLs) involving multiple viral gene products. Based on this, it has been suggested that a comprehensive analysis of the polymorphisms in HIV proteins is of value for understanding the virus transmission and pathogenesis as well as for the efforts towards developing anti-viral therapeutics and vaccines. This study, for the first time, describes an in-depth analysis of genetic variation in Vpr using information from global HIV-1 isolates involving a total of 976 Vpr sequences. The polymorphisms at the individual amino acid level were analyzed. The residues 9, 33, 39, and 47 showed a single variant amino acid compared to other residues. There are several amino acids which are highly polymorphic. The residues that show ten or more variant amino acids are 15, 16, 28, 36, 37, 48, 55, 58, 59, 77, 84, 86, 89, and 93. Further, the variant amino acids noted at residues 60, 61, 34, 71 and 72 are identical. Interestingly, the frequency of the variant amino acids was found to be low for most residues. Vpr is known to contain multiple CTL epitopes like protease, reverse transcriptase, Env, and Gag proteins of HIV-1. Based on this, we have also extended our analysis of the amino acid polymorphisms to the experimentally defined and predicted CTL epitopes. The results suggest that amino acid polymorphisms may contribute to the immune escape of the virus. The available data on naturally occurring polymorphisms will be useful to assess their potential effect on the structural and functional constraints of Vpr and also on the fitness of HIV-1 for replication.

## Introduction

Humoral and cellular responses have been implicated in controlling viral and bacterial infections in addition to the host's innate immune responses. This is, indeed, demonstrated in the context of HIV-1 infection [1-3]. Specifically, CTL responses against the virus have been shown to limit the virus replication at a low level in the infected individuals. This is evident in the inverse correlation of CTL responses vs. virus load observed in acutely infected individuals [4-6]. Utilizing the rhesus macaque/SIV infection model, a suppressive effect on virus replication was shown for CTLs [7]. However, the initial CTL responses are not able to contain the virus at a later stage, possibly due to the emergence of viral variants that evade the immune responses resulting in continued virus replication [8,9]. Hence, an understanding of the CTL escape variants of HIV is important both in natural viral infections and also in the context of vaccine-induced immunity for developing effective CTL based polyvalent vaccines for containing diverse HIV-1 strains [10]. This is an area of research which is actively being pursued by several investigators [11,12].

The genome of HIV-1 has been shown to code for two regulatory proteins (Tat and Rev) and four auxiliary proteins (Vif, Vpr, Vpu and Nef) in addition to the Gag, Pol, and Env structural proteins [13]. The regulatory proteins Tat and Rev are essential for virus replication. Rev is involved in the transport of genomic and partially spliced subgenomic mRNA from the nucleus to the cytoplasm [14]. Tat is known as an activator of transcription of viral and cellular RNA. Vif plays an important role in HIV-1 replication in peripheral blood mononuclear cells (PBMC). Specifically, Vif prevents hypermutation in the newly made viral DNA through its interaction with APOBEC3G [15,16]. Vpr is known for its incorporation into the virus particles. The interaction of Vpr with the Gag enables its incorporation into the virus particle. Vpr is a multifunctional protein and is involved in the induction of apoptosis, cell cycle arrest, and transcriptional activation [17]. Vpu plays a role in the particle release and degradation of CD4 [14,18,19]. The features of Nef include downregulation of cell surface receptors, interference with signal transduction pathways, enhancement of virion infectivity, induction of apoptosis in bystander cells, and protection of infected cells from apoptosis [20-24].

Based on the data reported so far, it is clear that HIV-1 employs multiple strategies to successfully replicate in the infected individuals [14,25,26]. The enormous genetic variation that is generated through errors of reverse transcriptase enzyme may provide a pool of variants to evade the host immune responses against the virus and also result in the emergence of drug resistant viruses during treatment. In addition, it is also likely that the immunosuppressive effects of HIV-1 encoded proteins may attenuate the host immune responses in favor of the virus.

Upon infection of target cells by the virus, viral proteins are synthesized for carrying out the functions related to the virus replication and also exert effect on specific host cell functions. In addition, viral proteins are also targeted to the proteosomal degradation pathway. This process results in the generation of peptides, which are then translocated to the ER through TAP and are presented on the cell surface in association with human leukocyte antigen (HLA) class I molecules. The genetic variability present in the coding sequences of the virus may result in viral proteins with alterations in the CTL epitopes, which may lead to defective processing, presentation or lack of recognition of the epitope by the reactive CTLs. This is the likely mechanism of the CTL escape by HIV-1 and other viruses. The presence of multiple CTL epitopes has been demonstrated in HIV-1 proteins including Gag, Pol, Vif, Vpr, Tat, Rev, Vpu, Env and Nef. Though the characterization of the epitopes with respect to the viral proteins is achievable in individual cases, such an analysis at a population level is difficult to carry out for the following reasons: i) HIV-1 exhibits high genetic variation in different regions of the genome. The extent of heterogeneity among circulating HIV-1 strains is described to be in the range of 20% or more in relatively conserved proteins and up to 35% for Env protein [11]. In addition, there is also extensive diversity among HIV-1 within a subtype, ii) There are multiple subtypes of HIV-1, and iii) There are variables at the HLA loci. On the other hand, this limitation can be overcome to some extent by utilizing alternative approaches where information about CTL epitopes and their variants can be inferred from the sequences available for HIV-1 [27-29]. The HIV sequence database has information about the viral isolates from different parts of the world. This information can be used as a source to assess the extent of naturally occurring polymorphisms and their potential impact on CTL epitopes. We hypothesize that mutations or alterations in the residues which are part of the CTL epitope in the Vpr molecule are likely to affect the epitope at multiple levels (processing and recognition of the epitope). Recently, studies have addressed this issue using full length or partial HIV-1 genome sequences [30]. This has prompted us to carry out a comprehensive analysis of the extent of variation at the amino acid level in the auxiliary gene product Vpr of HIV-1.

The underlying reasons for the selection of Vpr for a comprehensive analysis are the following: i) Vpr is a virion associated protein, ii) Vpr plays a critical role for the replication of virus in macrophages, iii) Vpr is a transcriptional activator of HIV-1 and heterologous cellular genes, iv) Vpr arrests cells at G2/M, v) Vpr induces apoptosis in diverse cell types, vi) Vpr exhibits immune suppressive effect, vii) Vpr is present in the body fluids as an extracellular protein, viii) Vpr is highly immunogenic, ix) Vpr is a small protein comprising only 96 amino acids and x) Structural information for the whole Vpr molecule is available through NMR [17,31-34]. These features enable a detailed analysis of the polymorphisms in Vpr with respect to CTL epitopes, structure-function of the protein, and fitness of the virus for replication.

In this study, we have analyzed the predicted amino acid sequences of Vpr from global HIV-1 isolates available through the HIV database. Specifically, the extent of genetic variation in Vpr in the form of polymorphisms at the individual amino acid level was comprehensively analyzed. Several of the amino acid polymorphisms were found to be part of the experimentally verified and predicted CTL epitopes. The location and nature of the variant amino acid were found to affect the CTL epitope considerably. Hence, our results provide a glimpse into the genetic footprints of immune evasion in Vpr.

## Materials and methods

The goal of our studies is to assess the nature and extent of polymorphisms at the level of individual residues in the Vpr molecule. The sequences considered here comprise Vpr sequences derived from all the major subtypes of HIV-1. The details regarding the subtypes and the number of sequences from each subtype are presented in Table 1 and are taken from the HIV database [35-38]. In addition, we have included Vpr sequences derived from HIV-1 positive long term non-progressors (McKeithen et al., unpublished data). It should be noted that we have also included Vpr from SIV isolated from chimpanzees, as this is likely the progenitor virus for HIV-1. Vpr sequences from the database were accessed in January of 2007. The deletions in the Vpr molecule were excluded from our analysis. The alignment of Vpr sequences (which is available from the authors upon request) was analyzed manually for variant amino acids at the level of individual residue in Vpr from global and distinct subtypes of HIV-1.

**Table 1:** Vpr sequences used for the analysis of amino acid polymorphisms

Subtype Designation	Number of Vpr Sequences
A	67
B	294
C	185
D	44
F1	6
F2	4
G	8
H	3
J	2
K	2
AE	45
AG	39
AB	3
Cpx	28
Others (includes DF, BC, CD, BG, 01B, A1C, A1D, A1G, etc)	198
O	39
N	3
Cpz	4
Unclassified	3

## Results

### Characteristics of Vpr sequences selected for this study

The alignment of Vpr sequences has enabled us to analyze the differences at the level of each residue from diverse HIV-1 isolates. A total of 976 Vpr sequences have been used for alignment. The polymorphisms, with respect to the length, have been noted in Vpr by several investigators [17,39]. As this may pose problem for our analysis, our alignment does not take into account both deletions and insertions. The Vpr alleles are from diverse subtypes and include 67, 294, 185 and 44 Vpr sequences representing subtype A, B, C, and D, respectively (Table 1). The O, AE, AG, and cpx groups represent 39, 45, 39 and 28 Vpr sequences, respectively. Since the Vpr sequences are derived from different sources such as viral RNA, cloned viral DNA and proviral DNA from tissues, we have not made attempts to classify them in our analysis.

### Amino acid polymorphisms in the predicted Vpr sequences

Recently, the structure of full length Vpr has been resolved by NMR [40]. According to this study, Vpr consists of a flexible N-terminal domain (amino acids 1–16), helical domain I (HI) (residues 17–33), turn (residues 34–37), helical domain II (HII) (residues 38–50), turn (residues 51–54), helical domain III (HIII) (residues 55–77), and a flexible C-terminal domain (residues 78–96). Based on this structure, the polymorphisms observed in Vpr are presented with respect to the individual domain.

### N-terminus of Vpr (residues 1–16)

The results presented in Table 2 regarding the N-terminal domain of Vpr show that all the residues excluding the initiator methionine are susceptible for alterations. The altered amino acids or polymorphisms at each residue are indicated as variant amino acids or substitutions. For convenience, we have used Vpr from NL4-3 proviral DNA as a reference sequence. The amino acid sequence of NL4-3 Vpr is similar to HIV-1 subtype B consensus Vpr except for residues 28(S), 77(Q) and 83(I). Interestingly, the residue 9, which is G, has only one variant amino acid. In an earlier study, it was noted that a change in residue 3 from Q to R was not associated with cytopathic effect [41]. In our analysis, variant amino acids H, L, M, and P were also noted for Q. Studies involving synthetic peptides corresponding to the N-terminus and also the full-length Vpr molecule have shown that the Vpr sequence (residues PHN) have the ability to form a γ-turn. The residue 15(H) exhibits eleven, residue 16 (N) shows ten and residue 14 (P) shows four variant amino acids. While residue 2 has two, residues 5 and 12 register three variant amino acids. Residues 3, 4, 6, 7, 8, 10, 11, and 13 contain multiple variant amino acids ranging from five to eleven. The N-terminal domain contains a total of 79 variant amino acids. Of these, non-conserved substitutions correspond to about 80% of the residues.

**Table 2:** The polymorphisms in the N-Terminus of Vpr (residues 1–16)

Residue	Residues in NL4-3 Vpr	Variant residue(s) noted in viruses of different clades	Number of variants
1	M	none	none
2	E	D, K	2
3	Q	H, L, M, P, R	5
4	A	D, F, I, L, N, P, S, T, V	9
5	P	L, Q, S	3
6	E	A, D, G, K, Q, S, V	7
7	D	E, G, H, N, V	5
8	Q	A, E, H, L, P, R	6
9	G	R	1
10	P	A, L, N, S, T	5
11	Q	A, E, S, P	4
12	R	E, G, K	3
13	E	A, D, I, G, Q, V	6
14	P	H, L, Q, S	4
15	Y	C, D, F, G, H, L, M, N, P, S, V	11
16	N	A, D, E, H, I, P, Q, R, S, T	10

The impact of the majority of the polymorphisms on Vpr functions is not clear. Substitution of alanine for proline at residue 5 and 10 showed less or increased virion incorporation of Vpr, respectively [42]. Similarly, substitution of alanine for residue 12 reduced the cell cycle arrest function of Vpr [43]. On the other hand, substitution at residue 13 and 14 showed an increase in cell cycle arrest [42,44]. Hence, the naturally occurring polymorphisms are likely to affect the functions of Vpr.

### Helical domain I (HI residues 17–33)

NMR studies of full length Vpr show that a region comprising the residues 17–33 adapt a helical structure. This was also predicted by several algorithms. The polymorphisms observed for the residues 17–33 are presented in Table 3. The characteristics of the residues with respect to the variant amino acids are the following: residues 18, 23 and 26 show two substitutions; residue 20 has three substitutions; residues 25, and 29 show four substitutions; residues 21, 24, 27, 30 and 32 show five substitutions; and residues 17, 22, and 31 register six substitutions and residue 19 has eight substitutions. Interestingly, residue 28 exhibits the highest number of substitutions and residue 33 has only one substitution. This domain exhibits a total of 80 variant amino acids and 61 of them are of non-conservative in nature.

**Table 3:** The polymorphisms in Helical Domain I of Vpr (residues 17–33)

Residue	Residues in NL4-3 Vpr	Variant residue(s) noted in viruses of different clades	Number of variants
17	E	A, D, G, Q, T, V	6
18	W	G, R	2
19	T	A, I, L, M, P, R, S, V	8
20	L	I, M, V	3
21	E	A, D, G, K, T	5
22	L	F, I, M, P, T, V	6
23	L	S, V	2
24	E	D, G, K, Q, R	5
25	E	A, D, G, K	4
26	L	F, I	2
27	K	I, M, N, Q, R	5
28	S	A, D, E, G, H, I, K, N, Q, R, T, V	12
29	E	D, G, Q, V	4
30	A	D, P, S, T, V	5
31	V	A, D, I, L, M, T	6
32	R	G, K, Q, T, W,	5
33	H	R	1

Several laboratories including ours have reported on the importance of residues in the helical domain I for Vpr functions. Substitution of a proline residue for glutamic acid (residue 17, 21, 24, 25, and 29) has a drastic effect on the stability, subcellular localization, and virion incorporation of Vpr [44-49]. The variant amino acids noted in this domain have the potential to destabilize and disrupt the function of Vpr. Similarly, substitution of alanine for leucine residue affected the stability and virion incorporation of Vpr [45,48,50-53]. Based on the studies reported, varying amino acid arginine for histidine at residue 33 will affect the subcellular localization and virion incorporation of Vpr [54].

### Interhelical domain I (residues 34–37)

This region is present between helical domains I and II and comprises only four residues. It has been shown that residues in this region have the ability to form a γ-turn. The naturally occurring polymorphisms in this region are presented in Table 4. Site-specific mutagenesis studies have shown an important role for residues in subcellular localization, cell cycle arrest, apoptosis and virion incorporation of Vpr [42,44,51,55,56]. Residues 34 and 35 show only three substitutions. On the other hand, residue 36 and 37 register 10 and 16 substitutions, respectively. The variant amino acids reach a total of 31 and 21 of them are of non-conservative in nature.

**Table 4:** The polymorphisms in the Interhelical Domain 1 of Vpr (residues 34 – 37)

Residue	Residues in NL4-3 Vpr	Variant residue(s) noted in viruses of different clades	Number of variants
34	F	L, V, Y	3
35	P	H, L, S	3
36	R	G, I, K, M, N, P, Q, S, T, W	10
37	I	A, D, E, G, H, K, L, M, N, P, Q, R, S, T, V, Y	16

### Helical domain II (residues 38–50)

Studies with peptide (1–50 amino acids) and full-length Vpr have shown that residues 38–50 correspond to helical domain II of Vpr. The naturally occurring polymorphisms corresponding to the residues in this region are presented in Table 5. The characteristics of the substitution are the following: residues 39 and 47 exhibit a single substitution; residues 43, 46 and 50 record two substitutions; residue 38 shows four substitutions; residues 42, 45 and 49 show five substitutions; and residues 40 and 44 have eight substitutions. Nine and thirteen substitutions were noted for residues 41 and 48, respectively. This domain contains 64 variant amino acids and non-conservative substitutions correspond to 41 residues. Several laboratories have carried out experiments addressing the role of residues in this region by utilizing site-specific mutagenesis. The alteration of hydrophobic residues severely affected the virion incorporation and transcriptional activation of Vpr [43,44,50,56].

**Table 5:** The polymorphisms in Helical Domain II of Vpr (residues 38 – 50)

*Residue*	*Residues in NL4-3 Vpr*	*Variant residue(s) noted in viruses of different clades*	*Number of variants*
38	W	C, F, T, Y	4
39	L	F	1
40	H	I, L, M, N, Q, R, T, Y	8
41	N	A, D, E, G, H, Q, R, S, W	9
42	L	C, I, F, M, V	5
43	G	E, R	2
44	Q	E, H, L, K, N, R, T, V	8
45	H	F, L, Q, W, Y	5
46	I	D, V	2
47	Y	H	1
48	E	A, D, G, H, I, K, N, Q, R, S, T, V, Y	13
49	T	H, N, M, S, Y	5
50	Y	H, S	2

### Interhelical domain II (residues 51–54)

This region is located between helical domains II and III. Of the four residues which are part of this domain, only the residue G51 has been shown to reduce G2/M cell cycle arrest through alanine substitution [44]. The naturally occurring polymorphisms corresponding to the residues in this region are presented in Table 6. The characteristics of the substitutions are the following: residue 54 shows two substitutions; residue 51 shows three substitutions; residue 52 shows four substitutions and residue 53 shows five substitutions. The variant amino acids reach a total of fourteen and the majority of them are non-conservative substitutions.

**Table 6:** The polymorphisms in Interhelical Domain II of Vpr (residues 51 – 54)

Residue	Residues in NL4-3 Vpr	Variant residue(s) noted in viruses of different clades	Number of variants
51	G	E, K, R	3
52	D	A, G, I, N	4
53	T	A, L, N, P, S	5
54	W	G, R	2

### Helical domain III (residues 55–77)

The presence of helical domain III has been demonstrated by NMR [40]. Several laboratories including ours have shown the importance of this domain for the function of Vpr. The naturally occurring polymorphisms noted for the residues in this region are presented in Table 7. The characteristics of the substitutions are the following: residues 56, 64, 65, 71 and 75 exhibit two substitutions; residues 69, 70, 72, 73 and 76 register three substitutions; residues 57, 66 and 68 show four substitutions; residues 60, 61 and 67 show six substitutions; residues 62 and 63 have seven substitutions; residue 74 has eight substitutions; residues 58, 59, and 77 exhibit ten substitutions; and residue 55 shows eleven substitutions. While the variant amino acids reach a total of 108, 65 of them are of non-conservative nature. This region comprises LXXLL motif which is important for subcellular localization and also influences the virion incorporation of Vpr [44,57-62]. Additionally the LXXLL domain is also involved in Vpr-GR interaction and its subsequent role in virus replication [63,64].

**Table 7:** The polymorphisms in Helical Domain III of Vpr (residues 54–77)

Residue	Residues in NL4-3 Vpr	Variant residue(s) noted in viruses of different clades	Number of variants
55	A	E, G, I, L, M, P, Q, R, S, T, V	11
56	G	E, R	2
57	V	A, L, M, W	4
58	E	A, G, I, K, L, M, Q, R, T, V	10
59	A	D, F, I, L, M, N, P, S, T, V	10
60	I	F, L, M, T, V, Y	6
61	I	A, L, M, T, V, Y	6
62	R	I, K, L, Q, S, T, W	7
63	I	F, L, M, S, T, V, Y	7
64	L	F, V	2
65	Q	H, R	2
66	Q	H, K, L, R	4
67	L	A, F, I, M, P, Q	6
68	L	I, M, P, R	4
69	F	L, S, V	3
70	I	A, T, V	3
71	H	L, Y	2
72	F	S, Y, L	3
73	R	G, S, T	3
74	I	F, H, L, M, N, S, T, V	8
75	G	K, R	2
76	C	G, S, Y	3
77	R	A, H, L, K, N, P, Q, S, T, W	10

### C-terminus of Vpr (residues 78–96)

The naturally occurring polymorphisms corresponding to the residues in the C-terminus of Vpr are presented in Table 8. The characteristics of the substitutions for the residues in this region are the following: residue 80 has only two substitutions; residues 78, 79, 82 and 92 have three substitutions; residues 81 and 90 have four substitutions; residues 91 and 96 have five substitutions. All of the other residues have substitutions ranging from six to thirteen. Of the 124 variant amino acids in this domain, 100 of them are of non-conservative nature.

**Table 8:** The polymorphisms in the Carboxy-Terminal Region of Vpr (residues 78–96)

Residue	Residues in NL4-3 Vpr	Variant residue(s) noted in viruses of different clades	Number of variants
78	H	L, R, Y	3
79	S	N, R, T	3
80	R	A, K	2
81	I	G, M, R, V	4
82	G	A, D, S	3
83	V	H, I, L, M, N, P, T, V	8
84	T	A, F, G, I, L, M, N, P, Q, S, V, W, Y	13
85	R	A, H, I, L, P, Q, T, V, Y	9
86	Q	E, G, H, M, P, R, S, T, V, Y	10
87	R	A, E, G, K, M, N, P, Q, S, T	10
88	R	A, E, G, I, S, T	6
89	A	D, E, G, I, L, N, P, R, S, T, V	11
90	R	G, I, N, S	4
91	N	D, H, I, K, S	5
92	G	A, E, R	3
93	A	D, F, G, L, M, N, P, S, T, V	10
94	S	D, E, F, G, H, N, R, V	8
95	R	A, D, G, I, K, P, S, T	8
96	S	F, P, T, V, Y	5

This domain contains multiple arginine and serine residues. It has been reported that the arginine residues are important for the cell cycle arrest and subcellular localization [65,66]. Vpr is known to undergo post-translational modification and the serine residues located at 28, 79, 94, and 96 positions of the protein serve as substrates for the phosphorylation [67]. Vpr, devoid of phosphorylation through site-specific mutagenesis, severely affects replication of HIV-1 in macrophages [68]. Residue 28 contains equivalent proportion of amino acids N (44%) and S (48%) and Vpr of SIV cpz contains N or T at this position. On the other hand, serine residues at 79, 94, and 96 are conserved in SIV cpz Vpr.

The naturally occurring polymorphisms for the whole Vpr molecule reach a total of 498 substitutions. The non-conservative variant amino acids correspond to 72%. It is important to note that all the residues in Vpr have the propensity to accept variant amino acids. The data presented here also reveal that the variant amino acids noted with respect to some residues are identical. These include residues 60(I), 61(I), 34(F), 71(H) and 72(F). We have carried out a detailed analysis of the variant amino acids noted in distinct subtypes (A, B, C, and D) of HIV-1. Such an analysis could not be carried out for several groups because of the limited information available regarding Vpr alleles. The data generated for subtype B Vpr alleles are presented in Tables 9, 10, 11, 12, 13, 14, 15. The analysis of subtype B involves a total of 275 Vpr alleles. As expected, the extent of polymorphisms in subtype B is less in comparison to the total polymorphisms noted with all the Vpr alleles. Interestingly, there are several residues that did not have any variant amino acids. These include residues 9, 18, 26, 34, 35, 38, 42, 46, 64, 66, and 79. On the other hand, the residues without variant amino acids in subtype C are different from that of subtype B except for 9, 26, and 64. In addition, the frequency of variant amino acids at the level of each residue was also determined for subtype B Vpr. The results indicate that the frequency of variant amino acids is low in most cases (0.4–1.1%) except for the residues 7, 19, 37, 41, 45, 55, 60, 63, 77, 80, 84, 85, 86, 89, and 93. Analysis involving a large number of Vpr alleles also showed frequency patterns consistent with the data presented in Tables 9, 10, 11, 12, 13, 14, 15. With respect to the N-terminus domain (Table 9), the residue 7 (D) has residue N substitution with a frequency of 6.2%. Also, while the reference Vpr allele has Y at position 15, which is the predominant amino acid (85%), the variant amino acid F occurs to a limited extent (6.9%). Similar scenario is also applicable to the residues 28, 77, and 83 (Tables 10 and 15). The residue R 80, which has been implicated in cell cycle arrest function of Vpr, exhibits substitution of A with a frequency of 5.1%.

**Table 9:** The frequency of variant amino acids in the N-Terminus of Vpr (Residues 1–16)

Residue	Residues in NL4-3 Vpr	Variant residue(s) noted in viruses of subtype B*
1	M	no change
2	E	D _(0.4)_
3	Q	H _(0.4)_, R _(1.8)_
4	A	D _(0.7)_, T _(0.4)_, V _(1.1)_
5	P	L _(0.4)_, S _(0.4)_
6	E	A _(1.1)_, D _(0.4)_, K _(1.1)_, Q _(0.7)_
7	D	N _(6.2)_, V _(0.4)_
8	Q	H _(1.1)_
9	G	no change
10	P	L _(0.4)_, S _(0.7)_
11	Q	A _(0.7)_, E _(0.4)_, P _(1.8)_, S _(1.8)_
12	R	K _(0.4)_
13	E	I _(0.4)_, Q _(1.1)_, V _(0.7)_
14	P	Q _(0.4)_, S _(0.7)_
15	Y	C _(0.4)_, D _(0.4)_, F _(6.9)_, H _(5.0)_, N _(0.7)_, S _(0.4)_, V _(0.4)_
16	N	A _(0.4)_, H _(1.1)_, I _(0.4)_, Q _(0.7)_, P _(0.4)_, R _(0.4)_, S _(0.4)_, T _(0.7)_

*275 Vpr alleles were used for analysis.

The numbers in the parentheses represent the percent frequency of the variant amino acid in the Vpr alleles analyzed.

**Table 10:** The frequency of variant amino acids in Helical Domain I of Vpr (Residues 17–33)

Residue	Residues in NL4-3 Vpr	Variant residue(s) noted in viruses of subtype B
17	E	A _(3.3)_, D _(0.4)_, G _(0.4)_, Q _(2.2)_, V _(0.4)_
18	W	no change
19	T	A _(12.7)_, R _(0.4)_
20	L	I _(4.4)_
21	E	G _(0.4)_
22	L	F _(0.4)_, I _(1.1)_, P _(0.4)_
23	L	V _(0.4)_, S _(0.4)_
24	E	G _(0.4)_, K _(0.4)_, Q _(0.7)_, R _(0.4)_
25	E	A _(0.4)_, D _(2.9)_, K _(0.4)_
26	L	no change
27	K	N _(0.4)_
28	S	G _(0.4)_, H _(0.7)_, N _(43.5)_, R _(4.7)_, T _(2.5)_
29	E	D _(0.4)_, V _(0.4)_
30	A	P _(0.4)_
31	V	A _(0.4)_, D _(0.4)_, I _(0.4)_, L _(0.4)_, T _(0.4)_
32	R	K _(3.6)_, Q _(0.4)_, W _(0.4)_
33	H	R _(3.6)_

**Table 11:** The frequency of variant amino acids in the Interhelical Domain 1 of Vpr (Residues 34–37)

Residue	Residues in NL4-3 Vpr	Variant residue(s) noted in viruses of subtype B
34	F	no change
35	P	no change
36	R	G _(1.1)_, W _(1.8)_, S _(1.5)_
37	I	A _(1.5)_, E _(3.6)_, G _(1.1)_, K _(0.4)_, L _(1.8)_, M _(2.5)_, N _(0.4)_, P _(16)_, R _(0.4)_, S _(0.7)_, T _(7.6)_, V _(19.3)_

**Table 12:** The frequency of variant amino acids in Helical Domain II of Vpr (Residues 38–50)

Residue	Residues in NL4-3 Vpr	Variant residue(s) noted in viruses of subtype B
38	W	no change
39	L	F _(0.4)_, I _(0.4)_
40	H	L _(2.2)_, N _(0.4)_, Q _(1.5)_, R _(0.4)_, T _(0.4)_, Y _(0.4)_
41	N	A _(0.7)_, D _(0.7)_, E _(0.4)_, G _(52.0)_, S _(30.5)_
42	L	no change
43	G	E _(0.4)_, R _(0.4)_
44	Q	R _(0.4)_
45	H	F _(0.7)_, L _(1.1)_, Q _(0.4)_, Y _(24.4)_
46	I	no change
47	Y	H _(0.4)_
48	E	A _(0.4)_, D _(2.5)_, G _(1.1)_, K _(0.4)_, Q _(0.4)_, V _(0.4)_
49	T	N _(0.7)_
50	Y	S _(0.4)_

**Table 13:** The frequency of variant amino acids in Interhelical Domain II of Vpr (Residues 51 – 54)

Residue	Residues in NL4-3 Vpr	Variant residue(s) noted in viruses of subtype B
51	G	E _(0.7)_, K _(0.4)_
52	D	N _(0.7)_, I _(0.4)_
53	T	A _(0.4)_, L _(0.4)_
54	W	R _(0.4)_, G _(0.4)_

**Table 14:** The frequency of variant amino acids in Helical Domain III of Vpr (Residues 55–77)

Residue	Residues in NL4-3 Vpr	Variant residue(s) noted in viruses of subtype B
55	A	E _(2.2)_, P _(1.1)_, Q _(0.4)_, T _(19.6)_, V _(1.8)_
56	G	R _(0.4)_, E _(0.7)_
57	V	W _(0.4)_
58	E	G _(1.1)_, I _(0.4)_, K _(1.1)_, Q _(0.7)_, V _(0.4)_
59	A	L _(0.4)_, P _(0.4)_, S _(0.4)_, V _(0.4)_
60	I	L _(16.4)_
61	I	L _(0.4)_, M _(1.1)_, T _(3.3)_, V _(1.5)_
62	R	K _(0.7)_, L _(0.4)_, S _(0.4)_
63	I	M _(5.8)_, S _(1.8)_, T _(11.3)_, V _(1.8)_
64	L	no change
65	Q	H _(0.4)_
66	Q	no change
67	L	M _(1.5)_, _P(0.7)_
68	L	M _(1.5)_
69	F	L _(1.8)_
70	I	T _(2.9)_, V _(1.1)_
71	H	L _(0.4)_, Y _(0.4)_
72	F	S _(1.5)_, Y _(0.4)_
73	R	T _(0.7)_
74	I	L _(0.4)_, M _(0.4)_, V _(0.4)_
75	G	R _(1.1)_
76	C	G _(1.1)_
77	R	H _(5.5)_, Q _(42.5)_

**Table 15:** The frequency of variant amino acids in the Carboxy-Terminal Region of Vpr (Residues 78–96)

Residue	Residues in NL4-3 Vpr	Variant residue(s) noted in viruses of subtype B
78	H	L _(0.7)_
79	S	no change
80	R	A _(5.1)_
81	I	G _(0.4)_, M _(0.7)_, V _(0.4)_
82	G	D _(0.7)_, S _(0.7)_
83	V	I _(86.9)_, L _(0.4)_, T _(0.7)_
84	T	A _(0.4)_, F _(0.4)_, G _(0.7)_, I _(30.9)_, L _(2.5)_, M _(0.4)_, N _(0.7)_, S _(0.4)_, V _(0.4)_
85	R	H _(0.4)_, I _(0.4)_, L _(2.5)_, P _(15.6)_, Q _(28.4)_, T _(0.4)_, Y _(1.1)_
86	Q	M _(0.4)_, P _(1.1)_, R _(21.1)_, S _(1.5)_, V _(1.1)_
87	R	A _(0.4)_, G _(1.5)_, K _(0.4)_, M _(0.4)_, N _(0.4)_, S _(3.3)_, T _(3.6)_
88	R	A _(2.2)_, G _(1.5)_, I _(0.4)_, S _(0.4)_, T _(0.7)_
89	A	E _(0.7)_, G _(0.4)_, P _(0.7)_, R _(2.2)_, S _(2.2)_, T _(10.2)_, V _(0.4)_
90	R	G _(0.4)_, N _(0.4)_, S _(0.4)_
91	N	D _(1.5)_, H _(0.4)_, I _(0.4)_, K _(0.4)_
92	G	A _(0.4)_, E _(0.4)_, R _(0.7)_
93	A	D _(0.4)_, L _(0.4)_, P _(0.7)_, S _(6.5)_, T _(2.2)_, V _(0.4)_
94	S	F _(0.4)_, G _(2.5)_, N _(1.1)_, R _(3.3)_, V _(0.4)_
95	R	A _(0.4)_, D _(0.4)_, I _(0.4)_, K _(0.4)_, T _(1.1)_, S _(0.4)_
96	S	P _(4.0)_

### Impact of amino acid polymorphisms on defined and predicted CTL epitopes in Vpr

It has been shown that a single amino acid change in the epitope enables the virus to evade the T cell surveillance [9,69]. Hence, it is of interest to analyze the polymorphisms in the context of both experimentally verified and predicted CTL epitopes. As Vpr is a highly immunogenic protein, several CTL epitopes have been already defined [12]. CD8+ epitopes are contiguous and nine amino acids long. The experimentally verified CTL epitopes in Vpr are presented in Table 16 with their location in the protein. We have presented the overall amino acid polymorphisms for each of the epitope. The experimentally verified CTL epitopes cluster in the region covering 1–70 residues of Vpr. The total amino acid polymorphisms range from 36 to 107 for the individual epitopes. For example, the CTL epitope comprising the residues REPHNEWTL contains 53 variant amino acids. Residues at position 1 to 9 of the epitope show 3, 6, 4, 11, 10, 6, 2, 8, and 3 variant amino acids, respectively.

**Table 16:** The extent of amino acid polymorphisms in experimentally defined CTL epitopes

Location of the epitope in Vpr	Amino acid sequence	Total number of variant amino acids in the CTL epitope	Reference
1 – 18	MEQAPENQGLQREPYNEW	87	[86]
9 – 26	GPQREPYNEWTLELLEEL	87	[87]
12 – 20	REPHNEWTL	53	[28,88,89]
19 – 28	TLEILEELKN	51	[86]
25 – 40	ELKNEAVRHFPRIWLH	87	[90]
29 – 37	EAVRHFPRI	52	[91-94]
30 – 38	AVRHFPRIW	52	[28,88,95]
31 – 50	VRHFPRWLHSLGQYIYETY	107	[96]
31 – 39	VRHFPRIWL	48	[97]
34 – 42	FPRIWLHGL	58	[28,87,89,97-103]
41 – 49	SLGQHIYET	49	[99]
41 – 57	GLGQYIYETYGDTWTGV	82	[87]
46 – 54	IYETYGDTW	36	[104]
48 – 57	ETYGDTWTGV	50	[87,97]
52 – 62	DTWAGVEAIIR	66	[97]
53 – 63	TWAVEAIIRI	69	[92]
55 – 70	AGVEAIIRILQQLLFI	86	[28]
59 – 67	AIIRILQQL	49	[10,28,89,96,98,105,106]
62 – 70	RILQQLLFI	38	[89,98,106]

In addition, we have also utilized bioinformatics approach to assess the effect of polymorphisms on CTL epitope . The predicted CTL epitopes with respect to several HLA class I alleles are presented in Table 17. The impact of polymorphisms on the CTL epitope was assessed by determining the estimate of half-time of disassociation of the molecule containing the epitope. For this purpose, we have considered 3, 1, 2, and 6 epitopes corresponding to HLA-A2, Cw-4, HLA B-7 and HLA B-2705, respectively. The influence of variant amino acids on the CTL epitope is presented in Table 18, 19, 20 with respect to HLA-A2 molecule. The epitopes considered for analysis correspond to residues 18–26, 38–46, and 66–74 of Vpr. While the reference peptide of the epitope located at residues 18–26 (Table 18) of Vpr shows the estimate of half time of disassociation value of 1213.356, the variant amino acid at position 1–9 in the epitope predicted a lower value. The substitution of variant amino acids at residue position 2 of the epitope affected the half-time value considerably. Interestingly, substitution of R lowered the value to 0.233. Similarly, the substitution of F for L at position 9 of the epitope also lowered the value to 4.233. The analysis of the epitope corresponding to the residues 38–46 is shown in Table 19. The variant amino acids at residue 39 and 41 drastically lowered the value. The residue 46 showed contrasting values based on the nature of the variant amino acid present. The impact of polymorphisms on the epitope corresponding to the residues 66–74 is shown in Table 20. The results show that both the location and nature of the amino acid have an effect on the half-time disassociation of the molecule, which may lead to defective processing, presentation, and recognition of the epitope.

**Table 17:** The predicted HLA Class 1 CTL epitopes in HIV-1 Vpr

Location of the predicted epitope	Amino acid sequence	HLA allele
7 – 15	DQGPQREPY	B62
8 – 16	QGPQREPYN	Dd
11 – 19	QREPYNEWM	B_2705
14 – 22	PYNEWMLDL	A24, Kd
18 – 26	WMLDLLEDL	A_0201, A_0205, B_2705, B_3901, Db_revised, Kd
26 – 34	LKHEAVRHF	Cattle_A20
31 – 39	VRHFPRPWL	B_2705
34 – 42	FPRPWLHEL	B7, Cw_0401
38 – 46	WLHELGQQI	A_0201
39 – 47	LHELGQQIY	B_3801
49 – 57	TYGDTWEGV	Kd
60 – 68	IVRTLQQLL	B7
61 – 69	VRTLQQLLF	B_2702, B_2705
64 – 72	LQQLLFVHF	B62, B_2705, B_3902
65 – 73	QQLLFVHFR	A_3101, B_2705, Cattle_A20
66 – 74	QLLFVHFRI	A_0201
72 – 80	FRIGCQHSR	B_2705, Cattle_A20
79 – 87	SRIGIIRGR	B_2705, Cattle_A20
87 – 95	RRGRNGSGR	B_2705, Cattle_A20

**Table 18:** Effect of variant amino acids on CTL epitope corresponding to residues 18–26 of Vpr

Amino Acid Sequence of Predicted Epitope	Score^β^
Prototype sequence (start position 18)^α^	
WMLDLLEDL	1,213.356
	
Natural variations observed at this epitope	
GMLDLLEDL	263.773
RMLDLLEDL	263.773
	
WALDLLEDL	23.334
WILDLLEDL	231.004
WLLDLLEDL	1,680.031
WPLDLLEDL	10.967
WRLDLLEDL	0.233
WSLDLLEDL	10.967
WVLDLLEDL	147.003
WTLDLLEDL	23.334
	
WMIDLLEDL	327.934
WMMDLLEDL	1,213.356
WMVDLLEDL	327.934
	
WMLALLEDL	295.940
WMLELLEDL	1,213.356
WMLGLLEDL	295.940
WMLKLLEDL	295.940
WMLTLLEDL	295.940
	
WMLDLSEDL	527.546
WMLDLVEDL	1,213.356
	
WMLDLLDDL	1,213.356
WMLDLLGDL	321.911
WMLDLLKDL	2,476.237
WMLDLLQDL	2,476.237
WMLDLLRDL	495.247
	
WMLDLLEDF	4.233
WMLDLLEDI	592.569

^α ^Accession No.: A1.TZ.01.A341_AY253314

^β ^Estimate of Half Time of Disassociation of a Molecule Containing This Epitope

**Table 19:** Effect of variant amino acids on CTL Epitope corresponding to residues 38–46 of Vpr

Amino Acid Sequence of Predicted Epitope	Score^β^
Prototype sequence (start position 38)^α^	
WLHELGQQI	196.763
	
Natural variations observed at this epitope	
CLHELGQQI	42.774
FLHELGQQI	196.763
TLHELGQQI	42.774
YLHELGQQI	196.763
	
WFHELGQQI	0.137
	
WLIELGQQI	196.763
WLLELGQQI	728.022
WLMELGQQI	728.022
WLNELGQQI	196.763
WLQELGQQI	196.763
WLRELGQQI	14.954
WLTELGQQI	196.763
WLYELGQQI	629.64
	
WLHALGQQI	47.991
WLHDLGQQI	196.763
WLHGLGQQI	47.991
WLHHLGQQI	47.991
WLHNLGQQI	47.991
WLHQLGQQI	47.991
WLHRLGQQI	47.991
WLHSLGQQI	47.991
WLHWLGQQI	47.991
	
WLHECGQQI	196.763
WLHEFGQQI	747.698
WLHEIGQQI	196.763
WLHEMGQQI	196.763
WLHEVGQQI	196.763
	
WLHELGEQI	96.414
WLHELGHQI	196.763
WLHELGKQI	196.763
WLHELGLQI	196.763
WLHELGNQI	196.763
WLHELGRQI	39.353
WLHELGTQI	196.763
WLHELGVQI	196.763
	
WLHELGQFI	1082.194
WLHELGQHI	196.763
WLHELGQLI	196.763
WLHELGQWI	1082.194
WLHELGQYI	1082.194
	
WLHELGQQD	0.281
WLHELGQQV	1311.751

^α ^Accession No.: A1.TZ.01.A341_AY253314

^β ^Estimate of Half Time of Disassociation of a Molecule Containing This Epitope

**Table 20:** Effect of variant amino acids on CTL Epitope corresponding to residues 66–74 of Vpr

Amino Acid Sequence of Predicted Epitope	Score^β^
Prototype sequence (start position 66)^α^	
QLLFVHFRI	223.888
	
Natural variations observed at this epitope	
HLLFVHFRI	7.612
KLLFVHFRI	783.608
LLLFVHFRI	380.609
RLLFVHFRI	223.888
	
QFLFVHFRI	0.155
QILFVHFRI	30.785
QMLFVHFRI	161.697
QPLFVHFRI	1.461
QQLFVHFRI	22.700
	
QLIFVHFRI	60.510
QLMFVHFRI	223.888
QLPFVHFRI	60.510
QLRFVHFRI	4.599
	
QLLFVLFRI	514.942
QLLFVYFRI	335.832
	
QLLFVHLRI	38.601
QLLFVHYRI	38.601
QLLFVHSRI	38.601
	
QLLFVHFRF	1.599
QLLFVHFRH	1.599
QLLFVHFRL	458.437
QLLFVHFRM	106.613
QLLFVHFRN	1.599
QLLFVHFRS	1.599
QLLFVHFRT	159.920
QLLFVHFRV	1,492.586

^α ^Accession No.: A1.TZ.01.A341_AY253314

^β ^Estimate of Half Time of Disassociation of a Molecule Containing This Epitope

## Discussion

Viral infections in individuals generally lead to a scenario where the virus is confronted by the host immune system involving both innate and adaptive immune responses. Regarding the latter, cellular and humoral immune responses have been shown to play a role in the control of infections of viruses including HIV-1 [70,71]. It has been suggested that an understanding of the correlates of protective immunity is an important requirement for the development of vaccines against HIV-1. Several studies have been published on this subject [71-73]. These studies point out a role for CD8+ and CD4+ T cell responses and neutralizing antibodies in the control of HIV-1 replication. For example, it has been reported that CD8+ cells control HIV-1 in the acutely infected individuals [4-6]. The relevance of CD8+ T cells for the control of virus infection was also shown in the case of SIV infected rhesus macaques [74,75]. Recently, the published data on CD8+ T cells in acute and chronic HIV-1 infection revealed that CTL epitopes are present in all of the proteins encoded by HIV-1. Virus replication, however, is not completely contained due to the emergence of CTL escape variant viruses. Based on this, it is suggested that vaccine efforts to control HIV-1 should take into account the high genetic variability noted among HIV-1.

The continued emergence of genetic variants is a characteristic feature of RNA viruses. RNA dependent RNA polymerase and reverse transcriptase are error-prone enzymes and have been implicated as a cause for the generation of variants [76,77]. The mutational changes in the protease and reverse transcriptase, depending on their location, may impact on their binding inhibitors targeting these enzymes. The viruses containing alterations may then be able to evade the inhibitory activities of the agents and are designated as drug-resistant variants. Similarly, the mutations in Env, Tat, and possibly other proteins can also evade the neutralizing antibody, CTL and T-helper cell responses [12,71]. The emergence of escape variants eventually repopulates the body in the face of immune responses against the virus. It has been suggested that immune escape may be a key step in the evolution of HIV-1 [30,78-80].

In an effort to understand the overall polymorphisms in a HIV-1 gene product, we undertook a comprehensive analysis of the predicted amino acid sequences of Vpr from diverse HIV-1 subtypes. Considering the genetic variation noted in diverse HIV-1 [39], our hypothesis is that the differences in Vpr and other viral proteins may enable the viruses to escape the host immunological pressures. To address this issue, we have initially compiled the polymorphisms in Vpr at the level of individual amino acid. Vpr contains only 96 amino acids. Hence, the small size of the protein is an advantage for a comprehensive analysis. For this purpose, we have turned to the Vpr sequences which are available in the HIV database and also sequences from specific groups such as HIV-1 positive long-term non-progressors. A total of 976 predicted Vpr amino acid sequences were used for our studies. The analysis revealed several characteristic features with respect to the individual amino acids in the Vpr. Of the 96 amino acids, all the amino acids except the initiator methionine have the propensity to change. This indicates that Vpr molecule is highly flexible in nature. The frequency of the variant amino acids, calculated for subtype B Vpr at the level of individual residue, revealed that substitution is very low for most of the residues. This suggests that many of the substitutions in Vpr may compromise the function and possibly the fitness of the virus. Interestingly, there are several amino acids that can accommodate ten or more alterations. We designate such amino acids as hot spots in Vpr which include residues 15, 16, 28, 36, 37, 48, 55, 58, 77, 84, 86 and 89. The underlying basis for the extensive genetic changes in specific regions of Vpr is not clear. It is likely that the error-prone reverse transcriptase, the secondary structure of RNA and other factors, either alone and/or in combination may play a role in the generation of genetic variants. In this regard, Yusim et al. [28] have noted that Integrase (IN) exhibits the least variability and Vpu exhibits the highest variability. Boutwell and Essex [27] also showed that the proportion of polymorphic amino acids ranged from a low of 55% (RT, IN) to a high of 94% (Vpu). In our analysis, Vpr variability is high which may likely be due to the inclusion of diverse isolates including the HIV-1 progenitor virus SIVcpz.

Vpr is known as a highly immunogenic protein. The presence of CTL epitopes verified through experimental approaches has been reported by several groups [12]. These include the region encompassing residues 9–70 of Vpr. Of the 96 residues, 62 (65%) have been shown to be associated with experimentally defined CTL epitopes. The data presented in Table 16 show that there are polymorphisms with respect to the experimentally verified CTL epitopes. The presence of variant amino acids at distinct locations within the epitope is likely to impact the CTL epitope. Further, we have also evaluated the effect of Vpr polymorphisms on CTL epitopes using the bioinformatics approach by calculating the estimate of half time of disassociation of the molecule containing the epitope. Such an analysis predicted several CTL epitopes all over Vpr including the C-terminus with respect to specific HLA class 1 molecules. The detailed analysis was carried out for different HLA alleles (HLA-A2, Cw-4, HLA-B7 and HLA-B2705) involving a total of 12 epitopes. The polymorphisms have also been analyzed for three predicted epitopes corresponding to residues 18–26, 38–46, and 66–74. The substitution of the variant amino acids for the residues comprising the epitope resulted in a drastic reduction in the value corresponding to the half time of the disassociation of the molecule containing the epitope. It should, however be noted that additional *in vitro *binding studies are necessary to confirm the predicted values.

Based on the data presented here, the amino acid polymorphisms noted in Vpr have the potential to contribute to the escape of the virus along with the epitopes present in other HIV-1 proteins [30]. It is also likely that the information regarding the polymorphisms at the CTL epitope will provide an opportunity to create an epitope-based vaccine that will exert control over viral isolates from different parts of the world. It is important to mention that the extensive HLA-associated amino acid polymorphisms noted here may also impact on the structure/function of Vpr and fitness of the virus [10,81-85]. The biological sources used for generating the sequence information of *vpr *include tissues from infected individuals, plasma viral RNA, and cloned viral DNA. For this reason, the Vpr sequences considered here for the analysis may be derived from both infectious and non-infectious viral genomes. Hence, there is a possibility that the amino acid polymorphisms noted here may or may not have a chance to be acted upon by CTL and T-helper cell pressures. It is known that amino acids in the proximal region of the epitope can also influence their immunogenic potential. The amino acid polymorphisms noted in the putative CTL epitopes can have an effect at a single and/or multiple levels in the generation of immune response: i) The mutations may eliminate the binding of the peptide to the appropriate HLA molecule, which will be presented on the cell surface. ii) Mutations may also disrupt the interaction with the T-cell receptors. iii) Mutations may disrupt the intracellular processing of the peptides. This results in the escape of the cells expressing the viral proteins from the surveillance of CD8+ T cells. The variant amino acids present in the proximal or far away from the epitope could influence through interference with the processing of the peptide from the protein. With regard to the latter, the variant amino acids may be either independent or compensatory in relation to changes in specific residues of Vpr. In addition, variant amino acids, which are part of overlapping epitopes presented by different HLA molecule, can also exert an influence on the epitope [30].

HIV variability is an important factor that should be taken into account in the efforts directed towards the development of vaccines against HIV-1. In order for the vaccines to be effective against diverse HIV-1, strategies that are being considered include consensus sequence approaches and polyvalent vaccines in the form of a mixture of genes/proteins from different subtypes of HIV-1. Despite the extensive variability reported for HIV-1, the nature and extent of variation has not been systematically investigated. Such an analysis is difficult to carry out for HIV-1 Gag, Pol or Env protein due to its size. It is for this reason that we have selected Vpr, a small protein. The results presented for Vpr here are interesting and novel as they describe genetic variation involving global HIV-1. Surprisingly, the frequency of the variant amino acids for most of the residues is low. This suggests that majority of the residues cluster around a sequence shared by HIV-1 isolates of different subtypes. It is likely that the influence of the residues on the fitness of the virus counters the variability, thus limiting the genetic variation. The information on Vpr polymorphisms will be of value for the development of vaccines based on the auxiliary genes of HIV-1.

## Authors' contributions

AS, VA, AK, AB, VS, RC and AC participated in the analysis of the predicted amino acid sequences of Vpr. SM, DD and BS provided information regarding the structure-function of Vpr. NM and RM contributed to the analysis of polymorphisms in Vpr from the structural angle. AS, VA, SM, VS, AC, and RC were involved in the preparation of the manuscript. All authors read and approved the final manuscript.
